# Intrinsic hierarchical structural imperfections in a natural ceramic of bivalve shell with distinctly graded properties

**DOI:** 10.1038/srep12418

**Published:** 2015-07-22

**Authors:** Da Jiao, Zengqian Liu, Zhenjun Zhang, Zhefeng Zhang

**Affiliations:** 1 Shenyang National Laboratory for Materials Science, Institute of Metal Research, Chinese Academy of Sciences, Shenyang 110016, China

## Abstract

Despite the extensive investigation on the structure of natural biological materials, insufficient attention has been paid to the structural imperfections by which the mechanical properties of synthetic materials are dominated. In this study, the structure of bivalve *Saxidomus purpuratus* shell has been systematically characterized quantitatively on multiple length scales from millimeter to sub-nanometer. It is revealed that hierarchical imperfections are intrinsically involved in the crossed-lamellar structure of the shell despite its periodically packed platelets. In particular, various favorable characters which are always pursued in synthetic materials, e.g. nanotwins and low-angle misorientations, have been incorporated herein. The possible contributions of these imperfections to mechanical properties are further discussed. It is suggested that the imperfections may serve as structural adaptations, rather than detrimental defects in the real sense, to help improve the mechanical properties of natural biological materials. This study may aid in understanding the optimizing strategies of structure and properties designed by nature, and accordingly, provide inspiration for the design of synthetic materials.

The mechanical properties of materials, when compositions or constituents are fixed, rely essentially on their structure^1^. The structural design and control have been an everlasting theme to achieve optimized properties in synthetic materials^2^,^3^,^4^. Nonetheless, it seems that some limitations are still extremely difficult to be circumvented by using empirical methods. For instance, as two important mechanical properties, strength and toughness invariably tend to be mutually exclusive in most synthetic materials^5^. As a result, generally a compromise has to be made between them to obtain a balanced combination. Besides, the formation of intended structure always necessitates stringent conditions which can be quite costly^3^. In this respect, nature has achieved great successes in designing high-performance materials using limited constituents at ambient conditions^4^. Natural evolution and selection over millions of years endow the biological structural materials with outstanding mechanical properties far exceeding those can be expected according to simple rule-of-mixtures from their components^6^,^7^,^8^. Such high mechanical efficiency is well exemplified by the mollusk shells of which the structure and mechanical properties have been extensively investigated^9^,^10^,^11^,^12^,^13^,^14^,^15^,^16^,^17^,^18^,^19^,^20^. Generally shells are composed of simple constituents of brittle minerals and weak biopolymers. Yet high strength and good toughness can be achieved simultaneously owing to the highly intricate hierarchical structure^9^,^10^,^11^,^12^,^13^,^14^,^15^,^16^,^17^,^18^,^19^,^20^, such as the typical laminated nacreous and crossed-lamellar arrangements of mineral platelets.

The structure of natural biological materials has attracted great attention because it may provide useful inspiration for the design of synthetic materials^4^,^18^. Indeed, a number of materials have been successfully synthesized through the bio-inspired design^3^,^18^,^21^. In particular, the laminated nacreous architecture of shells has been widely mimicked in exploiting high-performance structural materials^3^,^18^,^21^. Highly attractive mechanical properties can be obtained as a consequence. For example, by adopting freeze casting method, Munch *et al.*^21^ have developed a bulk hybrid ceramic-based Al_2_O_3_/PMMA composite with laminated structure similar to that of nacre. The fracture toughness can be significantly improved by ~300 times in energy term compared with its constituents accordingly.

So far, structural regularities have drawn the most attention in terms of both elucidating the structure of natural biological materials and designing bio-inspired synthetic materials^4^,^8^. The structure designed by nature has conventionally been described according to its regular patterns, such as the crossed-lamellar structure of mollusk shells, despite the considerable hierarchical complexities^22^,^23^,^24^,^25^. Previous structural models have also been proposed based on the regularities. Whereas this does facilitate a direct capture of main structural features, it seems quite different from the general scenario for synthetic materials, especially from the physical metallurgy perspective. It is widely accepted that the mechanical properties of synthetic materials depend indeed strongly on their local deviations from structural periodicity, i.e. irregularities or so-called defects^1^,^2^,^26^,^27^. Such imperfections on different length scales may affect the properties in distinct manners. Taking the metals and alloys for instance, the zero-dimensional point defects, such as vacancies and interstitial and substitutional atoms, are crucial for the diffusion transport process which is intrinsically associated with thermal, electrical and mechanical properties^1^,^27^. Meanwhile, the macroscopic plastic deformation is generally mediated by the motion of dislocations, i.e. one-dimensional imperfections. Accordingly, the mechanical properties can be effectively tailored by controlling the generation, interaction, and annihilation of dislocations^1^,^2^,^27^. In this respect, interfaces (such as grain boundaries, phase boundaries and twin boundaries) and inclusions, known as the two- and three-dimensional imperfections respectively, play an important role in strengthening materials by hindering the dislocation motion^1^,^2^. Yet they may also act as possible stress concentrators to promote crack initiation and propagation, leading to premature failure^1^. In this scenario, structural imperfections are invariably a widespread concern for both characterizing and designing synthetic materials. However, insufficient attention has been paid to the structural imperfections or defects in terms of natural biological materials, especially in experimental studies. Indeed, several kinds of irregularities have been identified in some organisms from literature, e.g. nanoscale voids and sheet-like defects within nacre tablets^28^,^29^. Nonetheless, there is still a lack of systematic understanding on the characters of structural imperfections, especially their possible relationships with mechanical properties, in natural biological materials. Indeed, the insights that can be gained for guiding the design and control of structural imperfections of synthetic materials depend strongly on this knowledge.

Here the structure of bivalve *Saxidomus purpuratus* shell, of which the structure and mechanical properties have been preliminarily examined by Yang *et al.*^23^,^30^, is systematically investigated from multiple length scales. Hierarchical imperfections are revealed to be intrinsically involved in the crossed-lamellar structure of shell despite its periodically packed platelets. Their relations with mechanical properties are further discussed. It is expected that the elucidation of naturally-occurring structural imperfections may help unravel new mechanisms for improving properties designed by nature, and accordingly, aid in the design of synthetic materials.

## Results

### Overall structure and mechanical properties

**Figure 1:**
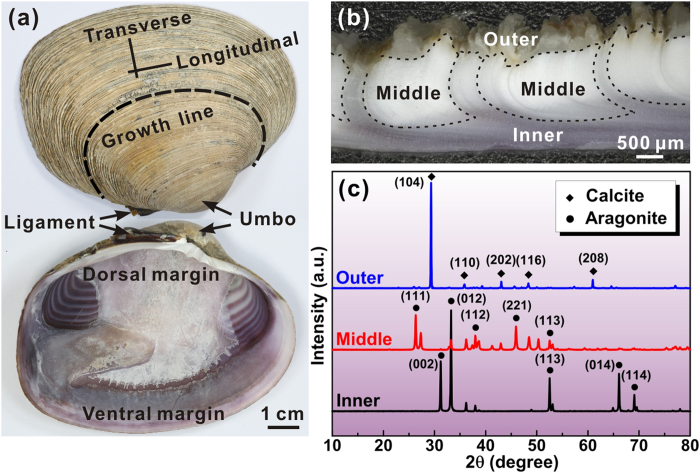
Macroscopic appearances and constituents of *S. purpuratus* shell. (**a**) Appearances of the exterior and interior surfaces of *S. purpuratus* shell. The longitudinal and transverse directions are denoted by the intersected lines. (**b**) Optical image of the transverse section. The boundaries between adjacent layers are denoted by dashed curves. (**c**) XRD patterns of the outer, middle and inner layers of shell.

Figure 1a shows the appearance of an *S. purpuratus* shell. The exterior surface appears tawny and manifests a series of concentric rings termed as growth lines. In comparison, the interior surface is quite smooth and characterized by a mixture of purple and white regions. These two colors are evenly arranged and parallel along the lateral side at the region near adductor muscle. As shown in Fig. 1b, the overall section can be primarily divided into three layers on the sub-millimeter scale throughout its thickness. The inner purple layer is covered with the white middle one which is further enveloped by a nearly transparent layer at the outmost surface. In particular, the inner layer may continuously extend across the whole thickness at some regions. The phase constituents of the three layers can be determined according to their X-ray diffractometer (XRD) patterns displayed in Fig. 1c. Whereas all the three layers are composed mainly of CaCO_3_ minerals, the crystal structure differs a lot among them, i.e. the outer layer is made up of calcite crystal while the constituents of both inner and middle layers manifest the aragonite structure. Moreover, the preferred orientations of aragonite crystals are markedly different between the inner and middle layers, as can be deduced from the relative intensities of diffraction peaks. It is noted that the outer calcite layer has been verified to be an intrinsic structure in the present *S. purpuratus* shell by examining more than ten shells. Indeed, it seems quite common that seashells possess an outer layer composed of calcite crystals although the majority of the inner of them are always in aragonite^14^,^15^,^17^,^19^,^31^. The outer calcite layer was not identified by Yang *et al.*^23^,^30^ maybe due to improper sample preparation because the calcite is quite brittle and can easily be detached from shell.

**Figure 2:**
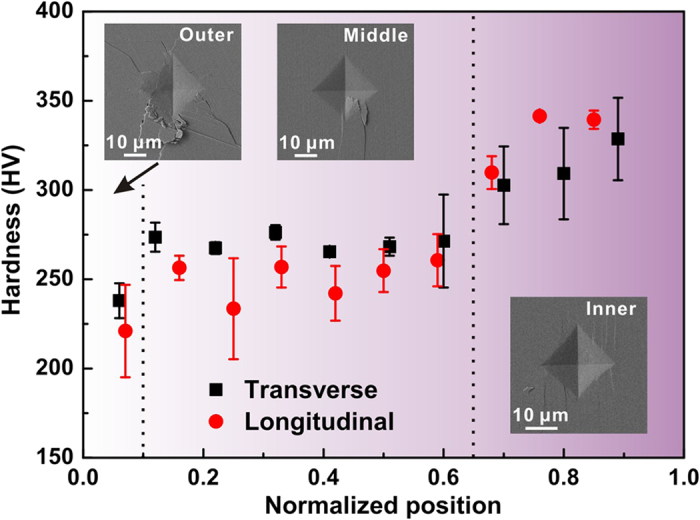
Indentation hardness and morphologies of *S. purpuratus* shell. Variations in Vickers hardness with the position along the outside-to-inside direction in transverse and longitudinal sections. Representative morphologies of indentation impressions corresponding to the outer, middle and inner layers are shown in the insets.

The mechanical properties of the three layers were preliminarily evaluated through Vickers hardness measurements. As illustrated in Fig. 2, the hardness increases almost monotonically in both the transverse and longitudinal sections along the outside-to-inside direction, which is consistent with the trend reported by Yang *et al.*^23^,^30^. Representative morphologies of indentation impressions corresponding to the three layers are displayed in the insets. It is seen that the outer layer demonstrates the most severe damage featured by abundant fractured blocks and long radial cracks surrounding the indentation residue. Prominent cracking also occurs at the corners of crater in the middle layer. These cracks manifest a low density, yet extend a long distance along a straight path, resulting in intensive damage concentration. In comparison, the inner layer is featured by plentiful microcracks oriented in certain directions and no obvious fractured blocks can be distinguished. This indicates a good damage tolerance of the inner layer in conjunction with its highest hardness.

**Figure 3:**
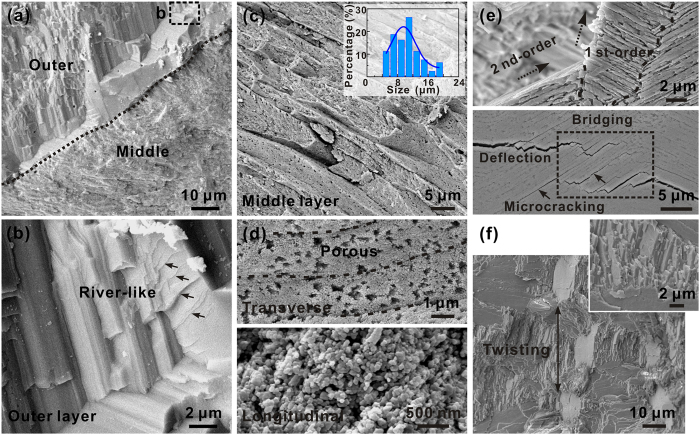
Structure of different layers of *S. purpuratus* shell. SEM micrographs of (**a**,**b**) outer and (**c**,**d**) middle layers of fractured *S. purpuratus* shell by manually bending. (**b**) manifests the magnified view of the circled regions in (**a**). The cross-sectional size distribution of constituent units for the middle layer is shown in the inset in (**c**). (**e**) Interactions between cracks and structure as well as (**f**) morphologies of fracture surface for the inner layer. The samples in (**e**) were etched in EDTA for 1 min.

The varying mechanical properties are also corroborated by the structure and fracture morphologies of the three layers. As shown in Fig. 3, representative river-like morphologies can be observed on the fracture planes of the outer layer, revealing the brittle transgranular cleavage of crystals^32^. The relatively low hardness and apparent brittleness of outer layer are expected to be closely related with its phase constituent of calcite. In comparison, the middle layer is constructed by highly porous and oriented units in cross-sectional dimensions of ~6–20 μm (Fig. 3c,d). Careful examination reveals that the constituent units are further made up by loosely packed nanoparticles in sizes of ~70–80 nm. The low packing density and weak unit boundaries may account for the low hardness and easy propagation of cracks in this layer. In contrast, the propagation of cracks follows a tortuous path and can be effectively hindered in the inner layer owing to its intricate structure. Multiple toughening mechanisms are introduced, e.g. crack deflection and twisting, uncracked ligament bridging, and microcracking, of which representative morphologies are displayed in Fig. 3e,f. The fracture surface also manifests a rather wavy feature. Considering that the middle and inner layers have the same phase constituent of aragonite, their distinct mechanical properties are supposed to originate essentially from the different structure. Although the performance of entire shell invariably comprise those of all components, the inner layer does possess the best combination of mechanical robustness and damage tolerance, and hence may contribute a lot to the mechanical properties. Useful inspiration may also be generated as a consequence for the design of synthetic materials. As a result, the structure of the inner layer has been mainly focused on and systematically elucidated in the following.

### Crossed-lamellar structure of inner layer at micrometer scale

**Figure 4:**
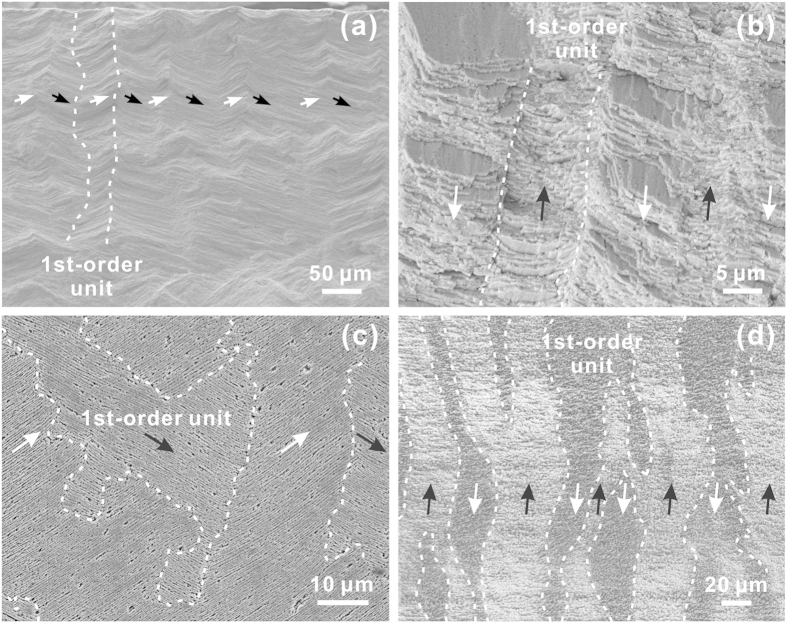
The first-order lamellae in the inner layer of *S. purpuratus* shell. SEM micrographs of the first-order lamellae in the inner layer for the (**a**,**b**) fractured and (**c**,**d**) etched specimens. (**a**,**c**) and (**b**,**d**) correspond to the longitudinal and transverse sections, respectively. The lamellar boundaries are indicated by dashed curves. The black and white arrows denote the different orientations of aragonite platelets in the lamellae. Samples in (**c**) and (**d**) were etched in EDTA for 1 and 5 min, respectively.

**Figure 5:**
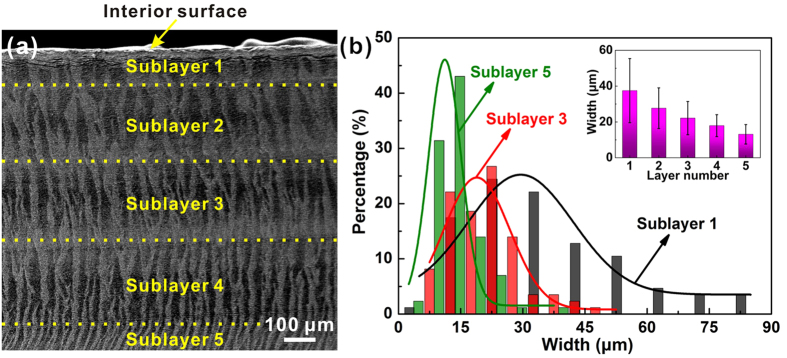
Dimensions of the first-order lamellae. (**a**) SEM image of the first-order lamellae in the transverse section of etched specimen and (**b**) representative lamellar width distributions in selected sublayers. The variation of lamellar width among different sublayers is shown in the inset.

Figure 4 shows the scanning electron microscope (SEM) micrographs of the inner layer along both longitudinal and transverse sections. A series of lamellae are alternatively arranged in a nearly parallel manner in the direction perpendicular to the interior surface. These lamellae, serving as the first-order structural units, are further composed of closely packed aragonite platelets with thickness of typically less than ~1 μm. In this scenario, the architecture can be classified as the crossed-lamellar structure, which has been commonly observed in bivalve shells^22^,^23^,^24^, according to the classification strategy proposed by Taylor^33^. Nonetheless, both the geometry and dimensions of the lamellae are extremely irregular, especially in the longitudinal section. The boundaries between adjacent lamellae are also quite tortuous as denoted by the dashed curves. The lamellae with different platelet orientations are supposed to be interconnected in three-dimensional space, as shown in Figs 4d and 5a. Furthermore, the width of lamellae also varies significantly depending on the position throughout the thickness. For the present case, the inner layer can be roughly divided into five sublayers from the interior surface downwards with a gradual decrease in the average lamellar width, as illustrated in Fig. 5. The size distribution of lamellae is quite inhomogeneous even within the same sublayers and tends to be more uniform with the increase in depth.

**Figure 6:**
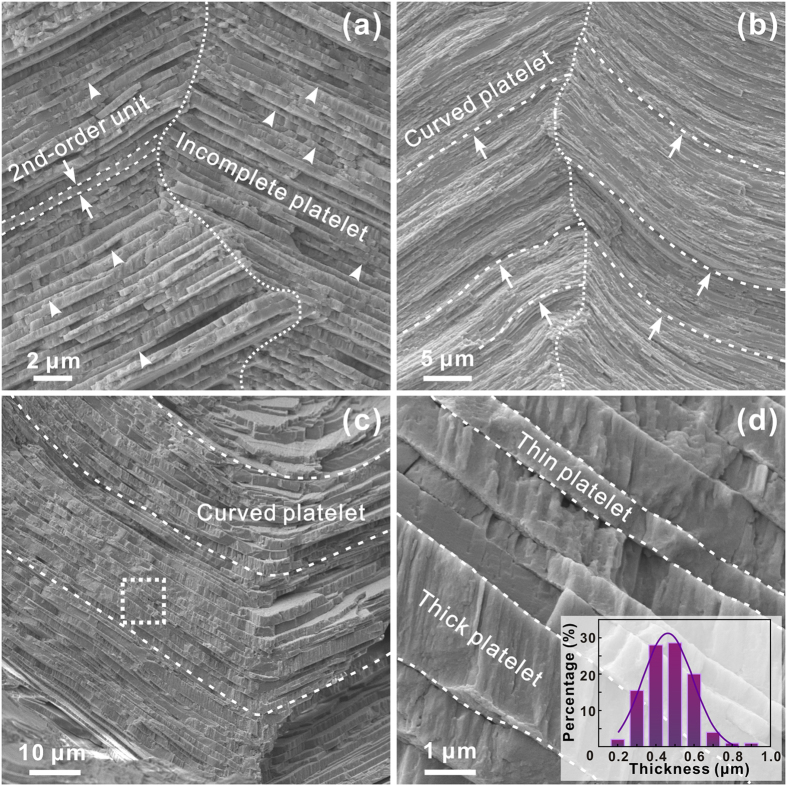
The second-order platelets in the inner layer of *S. purpuratus* shell. (**a**) shows the SEM micrograph of incomplete aragonite platelets. The curved platelets at varying degrees are shown in (**b**) and (**c**). (**d**) displays the magnified view of the circled region in (**c**). The distribution of platelet thickness is shown in the inset. The boundaries between first-order lamellae and second-order platelets are denoted by dotted and dashed curves, respectively.

The aragonite platelets which constitute the first-order lamellae can be seen as the second-order structural units. As shown in Fig. 6a, the platelets in adjacent lamellae are inclined by opposite angles with respect to the interior surface. They are intersected along curved boundaries between first-order lamellae with misorientations of ~90–150°. Careful examination reveals that a large number of incomplete platelets, which account for approximately ~12–13% of the total, are randomly distributed within the lamellae, as indicated by the arrowheads. This is supposed to contribute to a full space filling and dense packing to ensure the structural integrity. Meanwhile, the platelets *per se* are always deflected to varying extents even within the same lamellae, as displayed in Fig. 6b. In some cases, the platelets in neighboring lamellae can be heavily curved to form a continuous connection and smooth transition at their boundaries (Fig. 6c). In addition, the thickness of platelets manifests a wide distribution with the majority lying in the range of ~300–600 nm, as shown in Fig. 6d. The thickness of individual platelet can also be quite non-uniform at different positions along its length.

### Nanometer and sub-nanometer structure and imperfections

**Figure 7:**
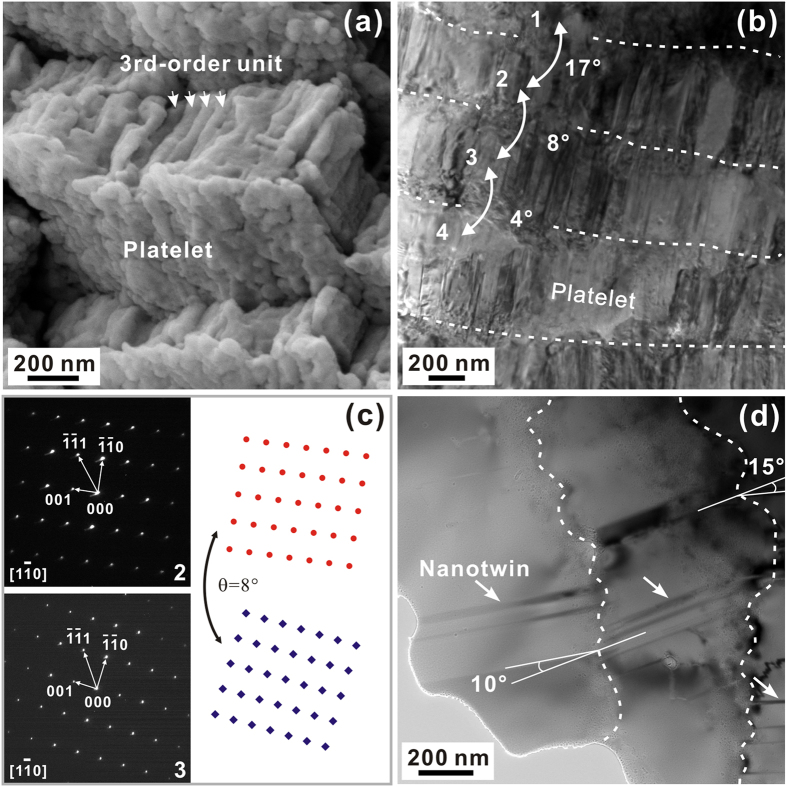
The third-order structural units in the inner layer. (**a**) SEM and (**b**) bright-field TEM micrographs of the third-order units in the aragonite platelets. The misorientations between adjacent platelets are depicted in (**b**). (**c**) SAED patterns of the platelets 2 and 3 marked in (**b**) and schematic illustration of their orientation relationship. (**d**) Direct bright-field TEM verification of the misorientation between neighboring platelets. Nanotwins are indicated by white arrows.

Figure 7 shows the fine structure within the second-order structural units. It is seen that the aragonite platelets are primarily composed of columnar rods with diameters of ~70–90 nm which are closely stacked in parallel. These rods can be seen as the third-order structural units for the inner layer of *S. purpuratus* shell. Besides, a thin granular layer can be distinguished at the ends of rods between neighboring platelets, which is analogous to the case of bivalve *Meretrix lamarckii* shell^24^. The aragonite structure can be further verified by the selected area electron diffraction (SAED) patterns, as illustrated in Fig. 7c. Whereas the rods within the same platelets are well aligned and possess identical crystallographic orientations, there exist varying low-angle misorientations of typically less than ~20° between adjacent platelets, as indicated in Fig. 7b–d. The platelet boundaries are also quite curved even down to the nanometer scale. Moreover, representative morphologies of nanotwins can be observed within the aragonite platelets, as indicated by the arrows in Fig. 7d. This implies that the adjacent rods may exhibit crystallographic twinning relations.

**Figure 8:**
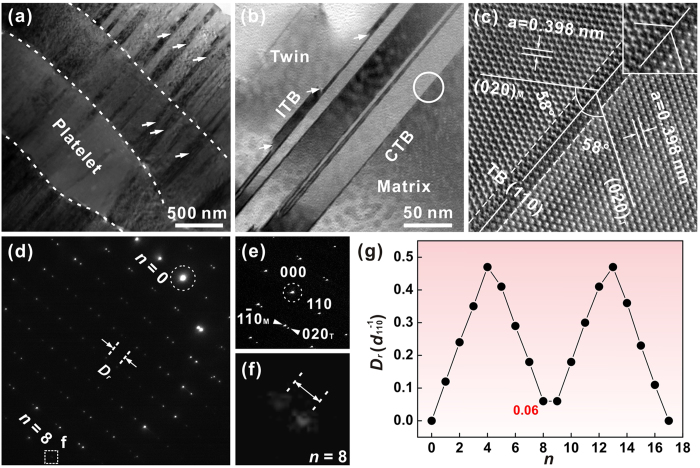
Nanotwins and twin boundaries in the aragonite platelets. Bright-field TEM micrographs of (**a**) nanotwins and (**b**) twin boundaries within the aragonite platelets. The ITBs are indicated by the arrows in (**b**). (**c**) High-resolution TEM micrograph of one CTB with the boundary region magnified in the inset. (**d**) SAED pattern and (**e**) corresponding index of the CTB denoted by the circle in (**b**). (**f**) Close view of the dotted box in (**d**). (**g**) Calculated relationship between the diffraction row number *n* and displacement of twin spots relative to matrix ones.

The twinning structure can be further clarified by the transmission electron microscope (TEM) analysis. As shown in Fig. 8a, nanometer scale twins with widths of ~30–90 nm are quite widespread within the aragonite platelets. These nanotwins tend to extend across the whole thickness of platelets and arrest at the platelet boundaries. They are parallel to each other within the same platelet, yet tilted slightly by varying degrees between adjacent platelets as a result of crystallographic misorientations. The twin boundaries manifest distinct morphologies of long straight lines and kink-like steps (Fig. 8b), which are characteristic of coherent twin boundaries (CTBs) and incoherent twin boundaries (ITBs), respectively. This is quite analogous to the cases observed in metals and alloys^2^,^26^, implying high similarities between the structure of biological and synthetic materials. Fig. 8c shows the high-resolution TEM image of one CTB. It turns out that the (020) symmetry planes with *d* = 0.398 nm in the matrix and twin are symmetrically tilted by 58° with respect to the (110) twining plane. This is consistent with the previous report about the twinning relation in another mollusk shell of *Pinctada maxima*^29^. It is noted that the crystal lattices on both sides of twin boundaries exhibit a slight mismatch, as magnified in the inset. This implies that the CTBs in this shell may not be perfectly symmetrical at the atomic level.

Figure 8d presents the SAED pattern composed of two sets of diffraction spots belonging to the matrix and twin at the CTB. The corresponding indexes are calibrated in Fig. 8e. It is seen that the displacement between matrix and twin spots changes in a regular manner along the [110] twining axis, i.e. initially increases and then decreases with the increase of the index *n*, as shown in Fig. 8d. The two sets of spots seem to overlap again when *n* = 8. Yet careful observation shows that there is still a slight distance between them (Fig. 8f). In order to calculate the period of overlapped spots, the conversion matrix *T* for twin has been used as below^34^,^35^:


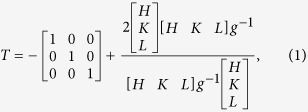


where (*HKL*) denotes the twinning plane and *g*^*−*1^ denotes the reciprocal metric which can be expressed as 
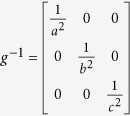
 for orthorhombic crystals (aragonite), respectively. Given an arbitrary lattice plane (*hkl*) in the twin coordinate system, the corresponding index in the matrix coordinate system (*h*^*t*^*k*^*t*^*l*^*t*^) can be calculated by the following equation^36^:


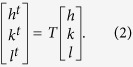


By plugging the lattice parameters of aragonite crystal, i.e. *a* = 0.49623 nm, *b* = 0.7968 nm, and *c* = 0.57439 nm (JCPDS file No. 41-1475^37^) into Eqs. (1) and (2), the displacement *D* of the twin spots relative to the matrix ones can be obtained as follows^38^:


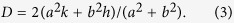


The displacement *D* includes two parts, i.e. the integer and the remainder (*D*_*r*_), wherein only the remainder can be manifested on the diffraction pattern^38^. The calculated values of *D*_*r*_ in different diffraction rows are shown in Fig. 8g. It is seen that the second superposition occurs at *n* = 17, prior to which the minimal value of *D*_r_ of 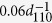 arises at *n* = 8 and 9 simultaneously. This conforms to the diffraction patterns of the (110) twin shown in Fig. 8d,f.

**Figure 9:**
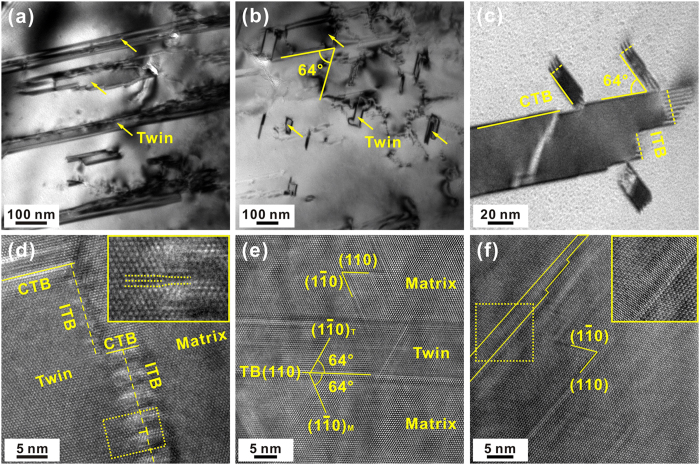
Nanoscale structural imperfections in the third-order units. Bright-field TEM micrographs of the (**a**,**b**) immature nanotwins and (**c**) coherent and incoherent twin boundaries. High-resolution TEM micrographs of (**d**) twin boundaries, (**e**) planar defects and (**f**) atomic dense band in the inner layer of *S. purpuratus* shell. CTBs and ITBs are denoted by solid and dashed lines, respectively.

Careful examination further reveals multiple irregular structural characters at the nanometer and sub-nanometer length scales. As shown in Fig. 9a,b, abundant immature nanotwins in smaller sizes are randomly distributed in the matrix. These nanotwins are featured by a high density of serrated ITBs. In particular, the ITBs at the ends of nanotwins demonstrate similar morphologies with those of the 9*R* structure observed in metallic materials^39^ which are caused by the introduction of interfacial dislocations (Fig. 9c). Indeed, partial dislocations can also be distinguished in the high-resolution TEM image of ITBs, as shown in Fig. 9d. The ITBs manifest as a smooth transition zone wherein the crystal lattices of matrix and twin are continuously interconnected. Whereas the nanotwins still prefer to grow along the (110) plane, secondary twinning plane with index of 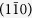 can also be identified (Fig. 9d). The secondary twins are tilted by an angle of 64° with respect to the primary ones. The nanotwins aligned along the two directions are intercrossed with each other. Meanwhile, a series of zigzag patterns, which are supposed to be caused by intersected stacking faults^40^, are distinguished on the high-resolution TEM images, as displayed in Fig. 9e,f. A dense band which appears like a new phase can also be observed embedding in the aragonite crystals (Fig. 9f). Although it is difficult to determine the crystalline structure definitely based on the present results, the atoms are shown to be more closely packed within the band. The above structural irregularities indicate huge complexities and widespread imperfections in the crossed-lamellar structure of shell even down to the atomic level.

## Discussion

**Figure 10:**
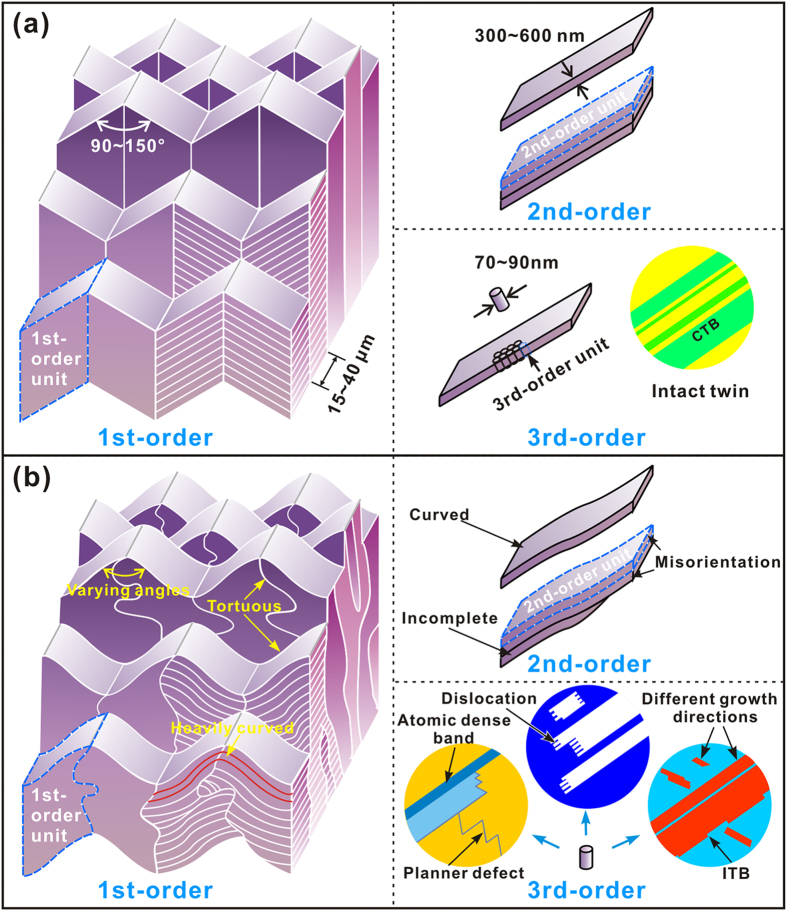
Schematic illustrations of crossed-lamellar structure of *S. purpuratus* shell. Illustrations of the inner layer structure (**a**) in the conventional manner and (**b**) following the modified scheme by taking the hierarchical structural imperfections into account.

The structure of natural biological materials has been widely explored for many organisms, especially for the mollusk shells^9^,^10^,^11^,^12^,^13^,^14^,^15^,^16^,^17^,^18^,^19^. In conventional practice, the structure has always been characterized according to the representative orderings or regularities^12^,^25^. Most existing structural models have been proposed following such manner for easily sketching and directly capturing the main structural features. In this scenario, the inner layer of present *S. purpuratus* shell can be described according to the typical crossed-lamellar structure containing periodically packed platelets and intact nanotwins, as can be schematically illustrated in Fig. 10a. However, as elucidated above, there indeed exist various structural imperfections on multiple length scales from millimeter to sub-nanometer. Firstly, the constituent units exhibit wide distributions of dimensions at all the three structural orders. Their shapes are irregular and many of them are even incomplete, immature, or heavily curved. Secondly, the orientations of interconnected constituents invariably manifest varying degrees of inconsistencies, as can be exemplified by the tilting angles between platelets in adjacent first-order lamellae and low-angle misorientations between nanotwins in neighboring aragonite platelets. The boundaries between constituents are also quite tortuous. Thirdly, the structure is even not perfectly ordered down to the atomic level. This can be clearly manifested by various irregularities, such as the immature nanotwins aligned in different orientations, ITBs and lattice mismatch on CTBs, partial dislocations, and planar defects. The above hierarchical imperfections are intrinsically involved in the crossed-lamellar structure of *S. purpuratus* shell. Nonetheless, it seems that these characters have not attracted much attention and cannot be reflected according to the conventional schemes. Based on the experimental results, modified schematic illustrations can be outlined by taking the imperfections into account, as shown in Fig. 10b. This is expected to give a better elucidation on the ingenious structure developed by nature in a more practical manner.

The structural imperfections are supposed to be intrinsic results of the growth of inorganic crystals through biologically-controlled self-assembly. They may form as a result of the variations in physiological conditions, such as temperature, the concentration of mineral ions and regulating organic proteins, which would fluctuate with time and sites during the process of biomineralization. Indeed, similar structural imperfections can also be found in other shells, such as *Strombus gigas* conch, of which the results are not presented here. It is widely accepted that the structure is tightly associated with the particular functions in natural biological materials and has been artfully adapted to achieve optimized properties^1^,^8^. From a critical structure-property connection perspective^1^,^8^, it is supposed that the hierarchical imperfections may not always act as “defects” to be detrimental to mechanical properties. Instead, they may stimulate multiple mechanisms to strengthen and toughen the materials. Firstly, the irregular dimensions and shapes of each order constituent units, such as the incomplete aragonite platelets, are favorable to ensure the structural integrity in eliminating possible pores and flaws in critical sizes^23^. This helps avoid the easy cracking in these stress concentrators^1^. It has also been revealed that the curvature or waviness of platelets could generate progressive interlocking during platelet sliding and propagation of inelastic deformation over large volumes in nacre^41^. The toughness can be enhanced effectively as a consequence. Secondly, the tortuous boundaries and misorientations between adjacent structural units can help stimulate toughening mechanisms of crack deflection and twisting and hence retard fracture^12^,^13^, as verified by the uneven propagation paths shown in Fig. 3e,f. Also, the interfaces can be strengthened by increasing the interfacial areas through the curved boundaries^1^. Thirdly, the mechanical properties could also benefit from the imperfections at the nanometer scale and down to the atomic level. It has been proven that introducing nano-scale CTBs is effective for achieving synchronous strengthening and toughening in synthetic materials of both metals and ceramics^2^,^42^. Meanwhile, the motion of partial dislocations has been revealed to facilitate the migration and twinning/detwinning processes of ITBs which favor improved mechanical properties^39^. It is supposed that the mechanisms generally concluded from ductile metals and alloys can also be extended to the inorganic biominerals in spite of their differences. For instance, dislocation emission and deformation twinning have been found in nacre and revealed to benefit the mechanical properties in similar manners with the cases in synthetic materials^20^. Dislocations and nanotwins have also been reported in other shells with their contributions to properties been clarified^6^,^14^,^20^ Therefore, the designing principles unraveled in synthetic materials have been abundantly adopted in the present shell. In this scenario, it is reasonable to assume that the structural imperfections in natural biological materials may serve as perfect adaptations designed by nature for their particular functions, rather than detrimental defects in the real sense.

It is noted that the organic components, though generally accounting for less than 5%, also play an important role in toughening the mollusk shells^43^,^44^,^45^. Nonetheless, the structure of shells is mainly dominated by the arrangement of inorganic minerals^4^,^18^. The organic components locate mainly at the interfaces between mineral platelets or lamellae. Thus it is reasonable and convenient to depict the structure of shells by examining the inorganic minerals. Also, it is difficult to distinguish the hierarchical structure of organic components experimentally. Before closure, it is worthwhile to highlight the possible implications of structural imperfections in both understanding the structure and properties of natural biological materials and developing bio-inspired synthetic materials. The overall mechanical properties are always dominated by the structural defects or imperfections in synthetic materials^1^,^2^,^42^. The characterization, design and control of imperfections act as one central theme of physical metallurgy^26^,^27^. However, the imperfections have not attracted sufficient attention in the experimental investigations of natural biological materials. Here hierarchical imperfections are revealed to be intrinsically involved on multiple length scales in the crossed-lamellar structure of *S. purpuratus* shell. In particular, the favorable structural features normally pursued in synthetic materials, such as nanotwins^2^,^42^, low-angle misorientations^46^, and slidable ITBs^26^,^39^, have been successfully incorporated. On the one hand, the elucidation of imperfections may aid in unraveling new strengthening and toughening mechanisms maybe unexpected so far yet has long been utilized by nature. On the other hand, the strategies developed by nature may be further applied to guide the design of synthetic materials to improve their properties.

In summary, the *S. purpuratus* shell can be divided into three layers throughout its thickness among which the inner layer possesses the best combination of high hardness and good damage tolerance. Despite the typical crossed-lamellar architecture consisting of three orders of structural units, there exist intrinsic hierarchical irregularities or imperfections on multiple length scales from millimeter to sub-nanometer in the inner layer. The structural units manifest broad distributions of dimensions and irregular shapes as well as varying misorientations and tortuous boundaries between each order. The structure is not perfectly symmetrical or periodical even down to the atomic level. These imperfections are supposed to contribute to improved mechanical properties rather than always to be detrimental which is analogous to the cases in synthetic materials. By highlighting the structural imperfections in natural biological materials, this study may help understand the structure and mechanical properties as well as mechanisms designed by nature from the physical metallurgy perspective, and also aid in the design of bio-inspired synthetic materials.

## Methods

The *S. purpuratus*, which inhabits the northwestern Pacific Ocean, is a marine bivalve mollusk in the family Veneridae. Fresh shells of adult *S. purpuratus* were obtained from a local seafood market. The directions parallel and vertical to the growth lines of shells are denoted as the longitudinal and transverse orientations respectively, as shown in Fig. 1a. The phase constituents were determined by an X-ray diffractometer (XRD, D/max-2500PC, Rigaku) with a Cu target. The Vickers hardness across the thickness of shells was measured using a hardness testing machine (AMH43, LECO) under load of 100 gf with a dwelling time of 13 s. The indentation impressions were examined by scanning electron microscope (SEM). The structure was characterized using the optical microscope, SEM and transmission electron microscope (TEM).

For optical observations, bulk specimens were cut from shells along the transverse direction using a low speed diamond saw. The sectional surface was carefully ground using wet abrasive paper and then polished with increasing grade diamond suspensions to ~0.5 μm finish. The polished section was etched in 2 wt.% ethylenediamine tetraacetic acid (EDTA) for 5 min, cleaned with distilled water immediately, and dried in air. The sample was observed using a digital microscope (Keyence VHX-1000).

Two types of specimens were adopted for SEM observations: (a) fragments of shells fractured along designed directions by manually bending and (b) polished and etched sections. For the latter, specimens were cut from shells along both longitudinal and transverse directions and then ground and polished in the same way as that for optical observation. The etching time was varied from 1 to 5 min depending on the sample orientation to clearly expose the structure. Finally, all the specimens were coated with a film of gold, and then examined with a field emission Supra 55 LEO SEM at an accelerating voltage of 6 kV.

For TEM characterization, the specimens were manually ground to ~70 μm in thickness and polished to a mirror finish. They were ion thinned at 5 kV and 8° incident angle for 5 hours and then 4° incident angle for 30 min using a Precision Ion Polishing System (Model 691, Gatan). Prior to observation, the surfaces of specimens were coated with a thin film of amorphous carbon in order to protect them against electron beam irradiation. Finally, the specimens were observed using a field emission G2 F20 Tecnai TEM instrument at an accelerating voltage of 200 kV.

## Additional Information

**How to cite this article**: Jiao, D. *et al.* Intrinsic hierarchical structural imperfections in a natural ceramic of bivalve shell with distinctly graded properties. *Sci. Rep.* **5**, 12418; doi: 10.1038/srep12418 (2015).
